# Exploring the Core Bacteria and Functional Traits in Pecan (Carya illinoinensis) Rhizosphere

**DOI:** 10.1128/spectrum.00110-23

**Published:** 2023-06-13

**Authors:** Mengting Shi, Tao Qin, Zhitao Cheng, Dingwei Zheng, Zhenyang Pu, Zhengfu Yang, Kean-Jin Lim, Menghua Yang, Zhengjia Wang

**Affiliations:** a State Key Laboratory of Subtropical Silviculture, Zhejiang A&F University, Hangzhou, Zhejiang, China; b College of Animal Science and Technology, College of Veterinary Medicine, Zhejiang A & F University, Hangzhou, Zhejiang, China; c Key Laboratory of Applied Technology on Green-Eco Healthy Animal Husbandry of Zhejiang Province, Zhejiang Provincial Engineering Laboratory for Animal Health Inspection and Internet Technology, Hangzhou, Zhejiang, China

**Keywords:** pecan, rhizosphere, core microbiome, function traits

## Abstract

Pecan (Carya illinoinensis) and Chinese hickory (Carya cathayensis) are important commercially cultivated nut trees. They are phylogenetically closely related plants; however, they exhibit significantly different phenotypes in response to abiotic stress and development. The rhizosphere selects core microorganisms from bulk soil, playing a pivotal role in the plant’s resistance to abiotic stress and growth. In this study, we used metagenomic sequencing to compare the selection capabilities of seedling pecan and seedling hickory at taxonomic and functional levels in bulk soil and the rhizosphere. We observed that pecan has a stronger capacity to enrich rhizosphere plant-beneficial microbe bacteria (e.g., *Rhizobium*, *Novosphingobium*, *Variovorax*, *Sphingobium*, and *Sphingomonas*) and their associated functional traits than hickory. We also noted that the ABC transporters (e.g., monosaccharide transporter) and bacterial secretion systems (e.g., type IV secretion system) are the core functional traits of pecan rhizosphere bacteria. *Rhizobium* and *Novosphingobium* are the main contributors to the core functional traits. These results suggest that monosaccharides may help *Rhizobium* to efficiently enrich this niche. *Novosphingobium* may use a type IV secretion system to interact with other bacteria and thereby influence the assembly of pecan rhizosphere microbiomes. Our data provide valuable information to guide core microbial isolation and expand our knowledge of the assembly mechanisms of plant rhizosphere microbes.

**IMPORTANCE** The rhizosphere microbiome has been identified as a fundamental factor in maintaining plant health, helping plants to fight the deleterious effects of diseases and abiotic stresses. However, to date, studies on the nut tree microbiome have been scarce. Here, we observed a significant “rhizosphere effect” on the seedling pecan. We furthermore demonstrated the core rhizosphere microbiome and function in the seedling pecan. Moreover, we deduced possible factors that help the core bacteria, such as *Rhizobium*, to efficiently enrich the pecan rhizosphere and the importance of the type IV system for the assembly of pecan rhizosphere bacterial communities. Our findings provide information for understanding the mechanism of the rhizosphere microbial community enrichment process.

## INTRODUCTION

Plants thrive in association with diverse microbial communities, which are capable of impacting the growth, development, and health of plants (1–3). Rhizosphere, the interface between the roots and the soil, is a key microhabitat for the plant microbiota. The microbial community of the rhizosphere constitutes part of a complex food pool that utilizes the nutrients released by the plant (for example, exudates, border cells, and mucilage) that are the major driving forces in the regulation of microbial diversity and activity in the immediate vicinity of plant roots (2). The rhizo-bacterial populations are recruited primarily from the corresponding bulk soil (here, soil without direct root effects) which serves as a microbial reservoir for the rhizo-microbiome (4). Several studies regarding microbial rhizosphere communities have shown the key role of plant species in shaping the microbial community in the rhizosphere (5, 6). However, most of these studies have focused on the characterization of rhizosphere microbiomes in herbaceous and crop plant species, including *Arabidopsis* (7, 8), rice (9), millet (10), soybean (11), corn (12), barley (13), wheat (14), sugarcane (15), cucumber (16), *and grapevine* (17) by exploring the structure, functional genes, and factors that determine the assembly of the microbiome. The studies on the rhizosphere microbiome of trees are confined only to citrus (18) and poplar (19, 20). Although plant microbiota are highly diverse, not all of these microbes play functionally important roles in the biology of their host, particularly tree hosts. Thus, defining the core microbiome enables researchers to refine the focus on stable taxa with a greater likelihood of influencing the host phenotype.

Pecan (Carya illinoinensis) and Chinese hickory (Carya cathayensis) are important commercially cultivated nut trees of the genus *Carya* (family *Juglandaceae*) with high nutritional value and substantial health benefits (21, 22). It has been reported that pecan and Chinese hickory are phylogenetically closely related plants (21). However, interestingly, they exhibit significant differences in phenotypes when it comes to abiotic stress and development. Pecan is cultivated on six continents and is adapted to a wide range of climate types from mild to harsh conditions. It exhibits high resistance to multiple abiotic stresses (23) and is considered a saline-alkali-tolerant plant (24). In contrast, Chinese hickory has a narrow distribution, suggesting that hickory may lack tolerance to abiotic stresses, such as colder climate, heat, flooding, drought, and salinity (21, 25, 26). The rhizosphere microbiome participates in the responses to environmental conditions and allows plants to achieve optimal growth and development (4), such as improving tolerance to abiotic stress (27), defending the plant host against pathogens (28), and modulating the plant immune system to induce resistance (29). Recent studies suggest that the core microbiome is stable and persistent and is more likely to influence host plant phenotypes (30, 31). Thus, the core taxonomic and functional components of the pecan rhizosphere microbiome might be vital for its resistance to multiple abiotic stresses.

In this study, we conducted deep shotgun metagenomic sequencing of the rhizosphere and the corresponding bulk soil microbiota in seedling pecan and seedling hickory. We aimed to identify the core taxa and functional traits specifically driven by pecan. We also sought to learn the plant factors that promote the high enrichment of core bacteria in the pecan rhizosphere and the functional traits that may play a key role in the assembly of the rhizosphere bacterial communities.

## RESULTS

### Structure and function of pecan and hickory rhizosphere-associated bacteria.

To investigate the rhizosphere microbiome and functional characteristics of pecan and hickory, we grew both plants at the same site. After 6 months, we collected the rhizosphere and bulk soil samples of the pecan and hickory seedlings. We then performed deep shotgun metagenomic sequencing of both the rhizosphere and bulk soil samples to gain insights into the rhizosphere microbial communities and their associated functional traits. More than 195,505,672,050 bp of shotgun metagenomic sequences were generated, yielding an average of 12,219,104,503 paired-end reads (150 bp) for each sample. After the removal of low-quality reads, 189,902,528,186 bp of clean reads were obtained. A total of 13,941,523 open reading frames (ORFs) were predicted from the metagenomic sequencing data sets. *De novo* assembly of clean reads resulted in 6,945,332 nonredundant catalog unigenes, which were used for subsequent analysis. Taxonomic classification revealed that bacteria comprised the predominant domain (98.61%), with small fractions of archaea (0.51%), eukaryotes (0.49%), and viruses (0.21%) detected based on the annotated unigenes. Thus, we focused our study on the comparative analysis of the community and the functional traits of the bacteria.

**FIG 1:**
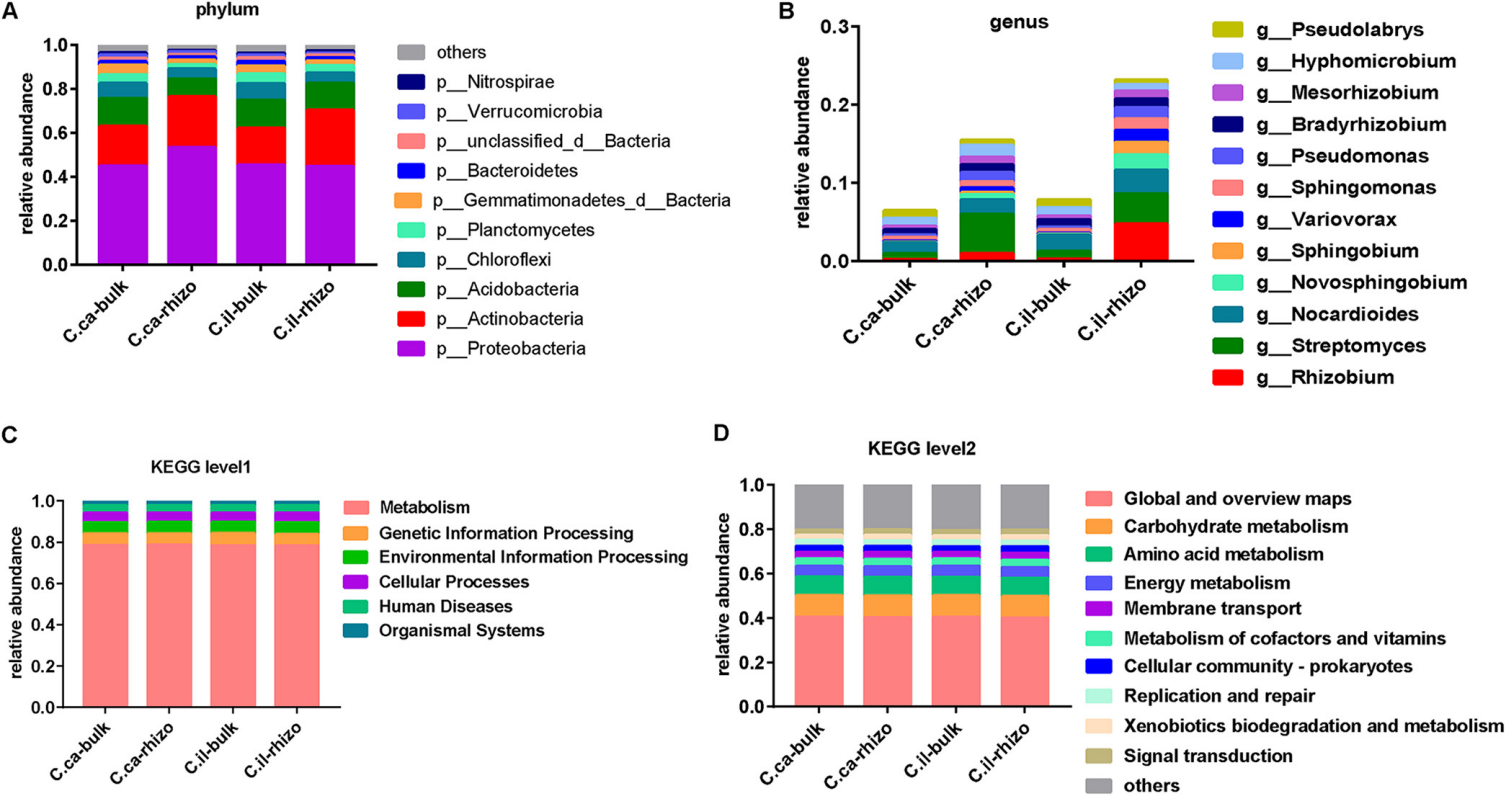
Structure and function of the seedling pecan and seedling hickory rhizosphere-associated bacteria. (A and B) Relative abundance of bacterial communities in rhizosphere and bulk soil samples. Community compositions are displayed at the phylum level (A) and the genus level (B). Genera with a total of <1% of samples were assigned as “others.” (C and D) Functional KEGG level 1 and KEGG level 2 pathway comparison of the rhizosphere and bulk soil bacteria. C.il_-bulk_, *Carya illinoinensis* (pecan)-bulk soil; C.il_-rhizo_, *Carya illinoinensis* (pecan)-rhizosphere soil; C.ca_-bulk_, *Carya cathayensis* Sarg (hickory)-bulk soil; and C.ca_-rhizo_, *Carya cathayensis* Sarg (hickory)-rhizosphere soil.

Taxonomic distributions of the rhizosphere microbiomes of the pecan and hickory plants revealed that a total of 153 genera were identified (see Data Set S1 in the supplemental material) at higher (phyla) taxonomic ranks. The dominant bacterial phyla in the pecan and hickory rhizosphere were *Proteobacteria*, *Actinobacteria*, and *Acidobacteria* (Fig. 1A). At the lower (genus) taxonomic ranks, a total of 3,376 genera were identified (Data Set S1). *Rhizobium*, *Streptomyces*, *Nocardioides*, *Novosphingobium*, and *Sphingobium* were the top five abundant genera in the pecan rhizosphere, while *Streptomyces*, *Nocardioides*, *Hyphomicrobium*, Pseudomonas, and *Bradyrhizobium* were the highly abundant genera (>1% relative abundance) in the hickory rhizosphere (Fig. 1B).

Besides providing taxonomic information, shotgun metagenomics also offers an opportunity to explore the functional diversity of the microbes recruited by plants (24). We utilized the Basic Local Alignment Search ToolP (BLASTP) of Diamond against the Kyoto Encyclopedia of Genes and Genomes (KEGG) Ortholog (KO) database to our assembled unigenes. We observed that 45% of the unigenes were assigned KO function annotation, with most of the KO annotated genes (67%) mapped to the KEGG pathways (see Data Set S2 in the supplemental material). In total, we identified 8,109 KOs from the unigenes. The gene functions associated with the KEGG pathway indicated that “metabolisms” were the predominant function in the total nonredundant protein-coding genes, followed by “genetic information processing,” and “environmental information processing” (Fig. 1C). “Carbohydrate metabolism,” “amino acid metabolism,” “energy metabolism,” “metabolism of cofactors and vitamins,” and “membrane transport” were detected as the most presented KEGG level 2 pathways (Fig. 1D).

### Seedling pecan has a strong selective power on rhizosphere bacteria and its associated function.

**FIG 2:**
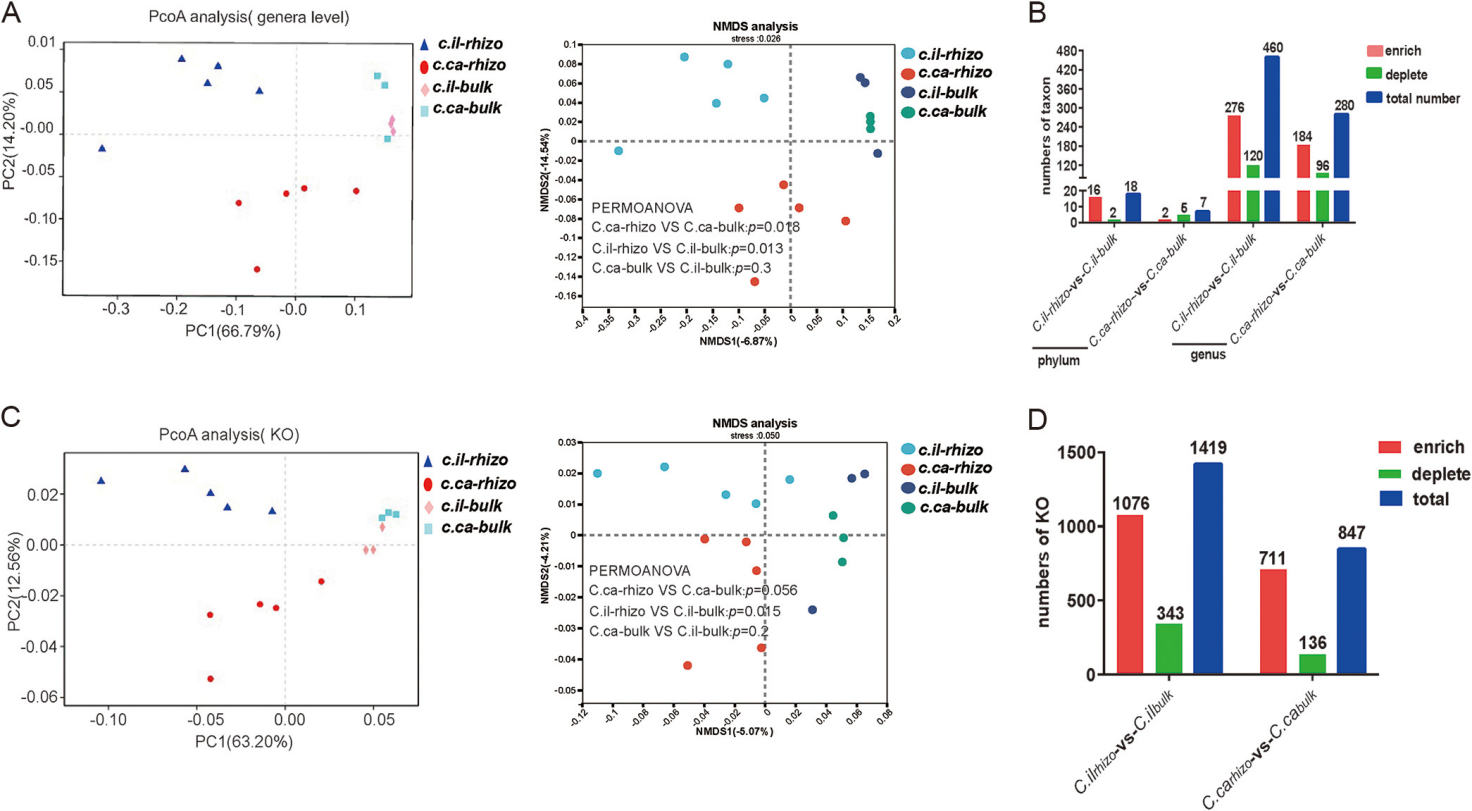
The seedling pecan has a strong selection on rhizosphere bacteria. (A and C) Principal coordinate analysis (PCoA) and nonmetric multidimensional scaling (NMDS) analysis of bacteria composition (A) and functional traits (C) based on the Bray-Curtis distance between the bulk soil and rhizosphere soil for each plant species using the metagenomic data. Differences in community or KO composition among C.ca-rhizo versus C.ca-bulk, C.il-rhizo versus C.il-bulk, and C.ca-bulk versus C.il-bulk groups were tested by PERMANOVA. (B) The number of the rhizosphere-enriched and the rhizosphere-depleted taxon at the phylum and genus level from bulk soil to rhizosphere in the pecan and hickory. (D) The number of the rhizosphere-enriched and the rhizosphere-depleted KOs from bulk soil to rhizosphere in the pecan and hickory. C.il_-bulk_, *Carya illinoinensis* (pecan)-bulk soil; C.il_-rhizo_, *Carya illinoinensis* (pecan)-rhizosphere soil; C.ca_-bulk_, *Carya cathayensis* Sarg (hickory)-bulk soil; and C.ca_-rhizo_, *Carya cathayensis* Sarg (hickory)-rhizosphere soil.

To determine whether the seedling pecan and seedling hickory had a selective power on the rhizosphere bacteria, we assessed the community composition of the bulk soil and rhizosphere samples using principal coordinate analysis (PCoA) and nonmetric multidimensional scaling (NMDS) analysis. The rhizosphere samples from the pecan and hickory plants were clustered separately, while the bulk soil samples were poorly clustered, indicating that pecan and hickory exerted selection on the bacterial community in the rhizosphere. Furthermore, the community composition in the pecan rhizosphere was different from that of the hickory rhizosphere (Fig. 2A; see Table S1 in the supplemental material). To further evaluate the selective power of the plant in the structuring of the bacterial rhizosphere community, we performed enrichment and depletion analyses at high (phylum) and low (genus) taxonomic ranks, based on the read abundance. We noted that 16 bacterial phyla were enriched and two phyla were depleted in the pecan rhizosphere microbiome, whereas five bacterial phyla were depleted and only two phyla were enriched in the hickory rhizosphere microbiome (Fig. 2B; see Data Set S3 in the supplemental material). Similarly, the enrichment analyses of the microbial genera revealed that 276 bacterial genera were enriched in the pecan rhizosphere microbiome and 120 were depleted (Fig. 2B; see Data Set S4 in the supplemental material), while 184 bacterial genera were enriched and 96 genera were depleted in the hickory rhizosphere microbiome (Fig. 2B; see Data Set S5 in the supplemental material). The total numbers of the enrichment and depletion of phyla or genera in the pecan rhizosphere were higher than those in the hickory rhizosphere (Fig. 2B). Our results indicate that the selective power of the pecan in the structuring of the bacterial rhizosphere community was stronger than that of the hickory.

The PCoA and NMDS, based on the Bray-Curtis distance (β-diversity), revealed significant differences in the functional trait composition of the rhizosphere and bulk soil bacteria (Fig. 2C; see Table S2 in the supplemental material), indicating that pecan and hickory apply selection in respect of the community composition of rhizosphere bacteria. To determine the plant selection in terms of shaping the function of the rhizosphere bacteria, we compared the KOs between the rhizosphere and bulk soil samples. Our findings indicate that 1,076 KOs were enriched in the pecan rhizosphere, whereas 406 KOs were depleted (Fig. 2D; see Data Set S6 in the supplemental material). In the hickory rhizosphere, 711 KOs were enriched and 136 KOs were depleted (Fig. 2D; see Data Set S7 in the supplemental material). Notably, the total numbers of the enrichment and depletion KOs in the pecan rhizosphere were higher than those in the hickory rhizosphere, indicating that the pecan is better at shaping the functional traits of rhizosphere bacteria. This intriguing finding has motivated us to explore the pecan rhizosphere microbiome in more detail.

### Core genera of the seedling pecan rhizosphere bacteria.

**FIG 3:**
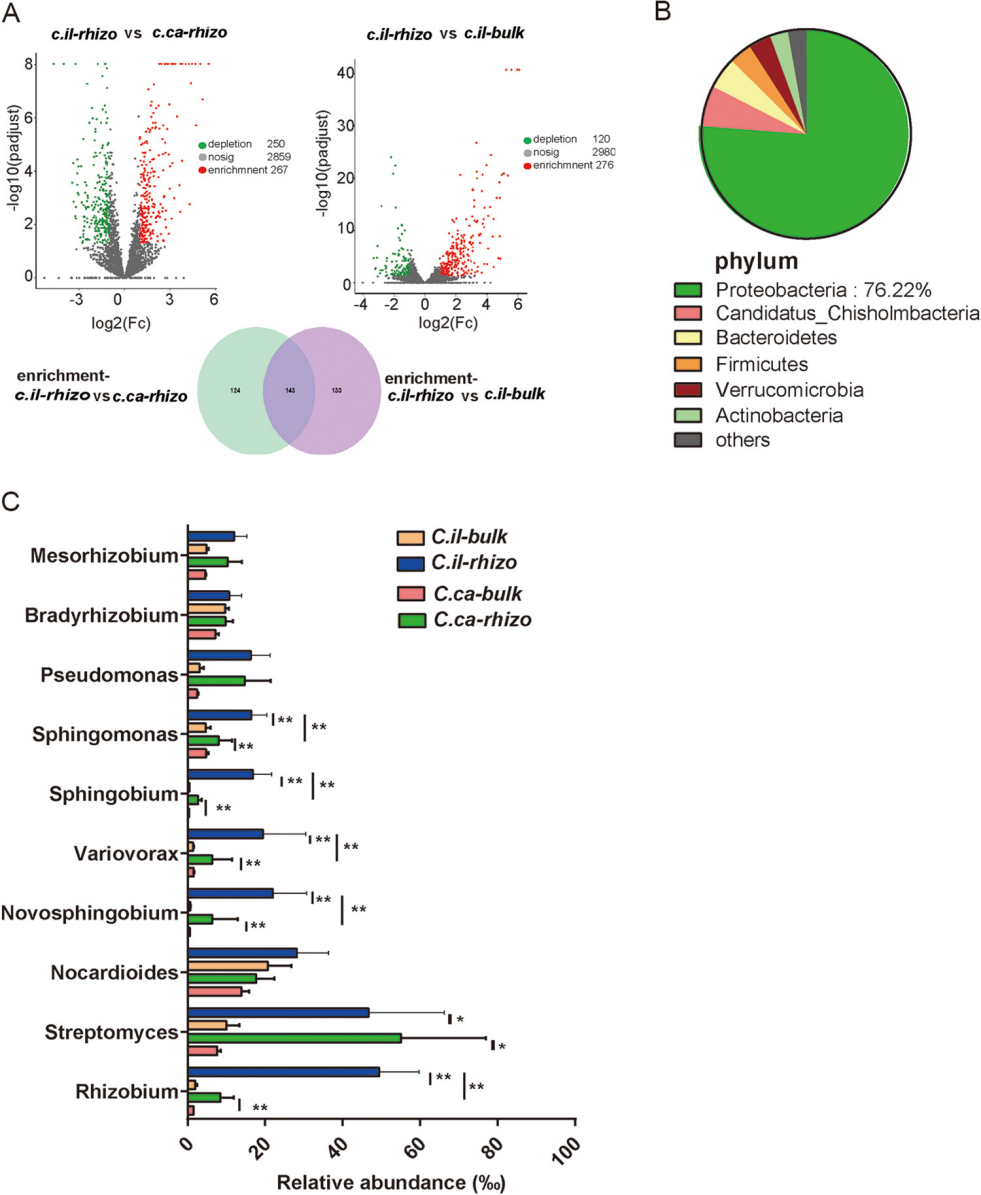
Core genera of the seedling pecan rhizosphere bacteria. (A) Volcano plots of the enrichment and depletion analysis on C.il-_rhizo_ versus C.il-_bulk_ and C.il-_rhizo_ versus C.ca-_rhizo_. Enrichment genera were indicated by red dots, depleted genera were indicated by green dots, and gray dots indicated no significant difference. The Venn diagram illustrates the unique and shared numbers of genera predicted from the enrichment genera data sets of C.il-_rhizo_ versus C.ca-_rhizo_ and C.il_-rhizo_ versus C.il_-bulk_. (B) The shared genera were annotated at phylum level, and then the relative abundance was present. (C) Average relative abundance (RA) ± SEM of the 10 most abundant genera in bulk soil and rhizosphere soil samples as revealed based on metagenomic data. **, *P* < 0.01; *, *P* < 0.05. C.il_-bulk_, *Carya illinoinensis* (pecan)-bulk soil; C.il_-rhizo_, *Carya illinoinensis* (pecan)-rhizosphere soil; C.ca_-rhizo_, *Carya cathayensis* Sarg (hickory)-rhizosphere soil.

Pecan exhibits high resistance to multiple abiotic stresses and yet hickory does not. We hypothesized that the higher abundance of genera in the pecan rhizosphere, in contrast to that in the bulk soil and the rhizosphere of hickory, probably plays a key role in enhancing pecan resistance to abiotic stresses. To identify the core general of the seeding pecan, we compared the genera in the seedling pecan rhizosphere with that in the corresponding bulk soil and the seedling hickory rhizosphere. The enrichment and depletion analyses were first performed between the bulk soil and rhizosphere soil of the pecan. Our analyses revealed that 276 genera were enriched in the pecan rhizosphere (Fig. 3A; Data Set S4). We then performed the enrichment and depletion analyses between the rhizosphere of the seedling pecan and the seedling hickory. The results indicate that 267 genera were enriched in the seedling pecan rhizosphere (Fig. 3A; see Data Set S8 in the supplemental material). To identify the core rhizosphere genera in the seedling pecan rhizosphere, we analyzed two enrichment data sets using a Venn diagram. Of these genera, 142 were identified as the core rhizosphere genera in the seedling pecan rhizosphere microbiome (Fig. 3A; Data Set S4). The majority of these genera (76.22%) belong to *Proteobacteria* (Fig. 3B; Data Set S4). We analyzed the abundance of the top 10 genera in the seedling pecan rhizosphere and observed that *Rhizobium*, *Novosphingobium*, *Variovorax*, *Sphingobium*, and *Sphingomonas* were significantly enriched in the seedling pecan rhizosphere compared with the corresponding bulk soil and the seedling hickory rhizosphere (Fig. 3C). This result suggests that these genera are core to the pecan and could be efficiently recruited by the seedling pecan.

### Core functional traits of the seedling pecan rhizosphere bacteria.

**FIG 4:**
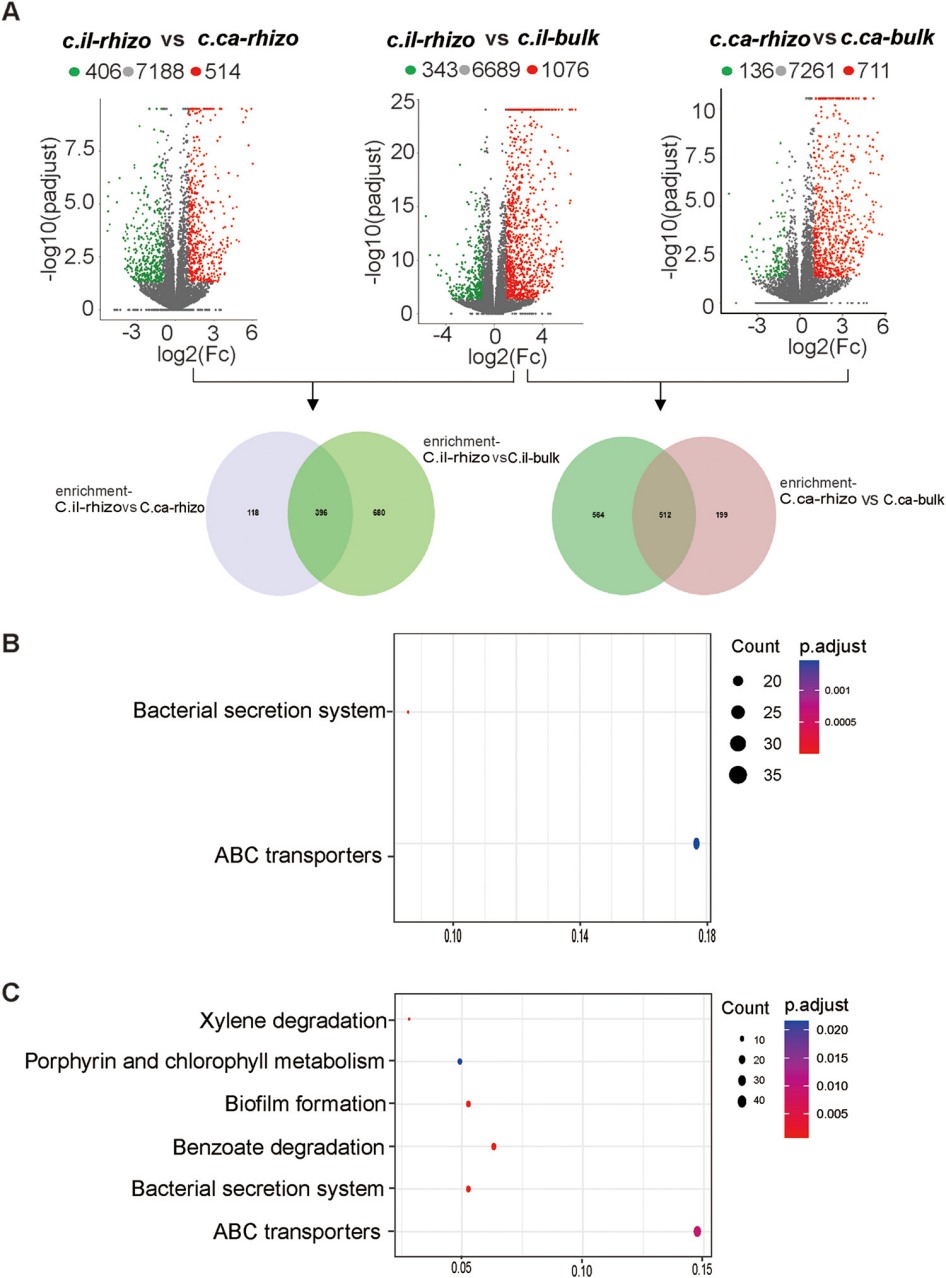
Core functional traits of the seedling pecan rhizosphere bacteria. (A) Volcano plots of the enrichment and depletion analysis on C.il-_rhizo_ versus C.ca-_rhizo_ (left), C.il-_rhizo_ versus C.il-_bulk_ (middle), and C.ca-_rhizo_ versus C.ca-_bulk_ (right). Enrichment KOs were indicated by red dots, depletion KOs were indicated by green dots, and gray dots indicated no significant difference. The Venn diagram illustrates unique and shared numbers of KO predicted from the enrichment KO data sets of C.il_-rhizo_ versus C.il_-bulk_ and C.il-_rhizo_ versus C.il-_bulk_ (left), C.il-_rhizo_ versus C.il-_bulk_, and C.ca-_rhizo_ versus C.ca-_bulk_ (right). (B) KEGG enrichment analysis of the shared KOs (the numbers of KO, 396) from the Venn diagram shown in A (left). (C) KEGG enrichment analysis of the specific KOs (the numbers of KO, 564) from the Venn diagram shown in A (right). The points represent the enrichment pathway; the size and the color of the points represent the numbers of KOs in the corresponding pathway and the *P* value of the KEGG pathway. Only the significantly enriched pathways (*P* < 0.05) were presented. C.il_-bulk_, *Carya illinoinensis* (pecan)-bulk soil; C.il_-rhizo_, *Carya illinoinensis* (pecan)-rhizosphere soil; C.ca_-bulk_, *Carya cathayensis* Sarg (hickory)-bulk soil; and C.ca_-rhizo_, *Carya cathayensis* Sarg (hickory)-rhizosphere soil.

To determine the core functional traits of the seedling pecan rhizosphere, we conducted enrichment and depletion analyses on the bulk soil pecan versus the rhizosphere soil pecan group (Fig. 4A, middle; Data Set S6) and the rhizosphere soil pecan versus the rhizosphere soil hickory group (Fig. 4A, left; see Data Set S9 in the supplemental material). We also generated Venn diagrams of the two enriched data sets (pecan-rhizo/hickory-rhizo enriched data set and pecan-rhizo/pecan-bulk enriched data set) to gain insight into the shared functional traits in the pecan rhizosphere. The results indicate that 396 KOs were shared in both data sets (Fig. 4A; Data Set S6), which were significantly enriched (*P* < 0.05) in the ABC transporter (ko02010) and bacterial secretion system pathway (ko03070) (Fig. 4B). To determine the specific functional traits of the pecan rhizosphere bacteria, Venn diagrams of pecan-rhizo-enriched (Fig. 4A, middle; Data Set S6) and hickory-rhizo-enriched (Fig. 4A, right; Data Set S7) data sets were performed. We observed that 564 KOs were specifically enriched in the seedling-pecan rhizosphere (Fig. 4A; Data Set S6). They were significantly enriched (*P* < 0.05) in the ABC transporter, bacterial secretion system, benzoate degradation, biofilm formation, porphyrin and chlorophyll metabolism, and xylene degradation pathway (Fig. 4C). These data indicate that the ABC transporter and the bacterial secretion system were the core functional traits of the seedling pecan rhizosphere bacteria.

### Linking the core functional traits and the species.

To visualize the association between the core functional traits and the taxonomy, we conducted a correlation analysis on the species and functional contribution in the seedling pecan rhizosphere samples. We extracted the taxonomic origin of the ABC transporter and the bacterial secretion system function and determined the contributions of the observed microbial taxa (genus level) in the pecan rhizosphere samples.

**FIG 5:**
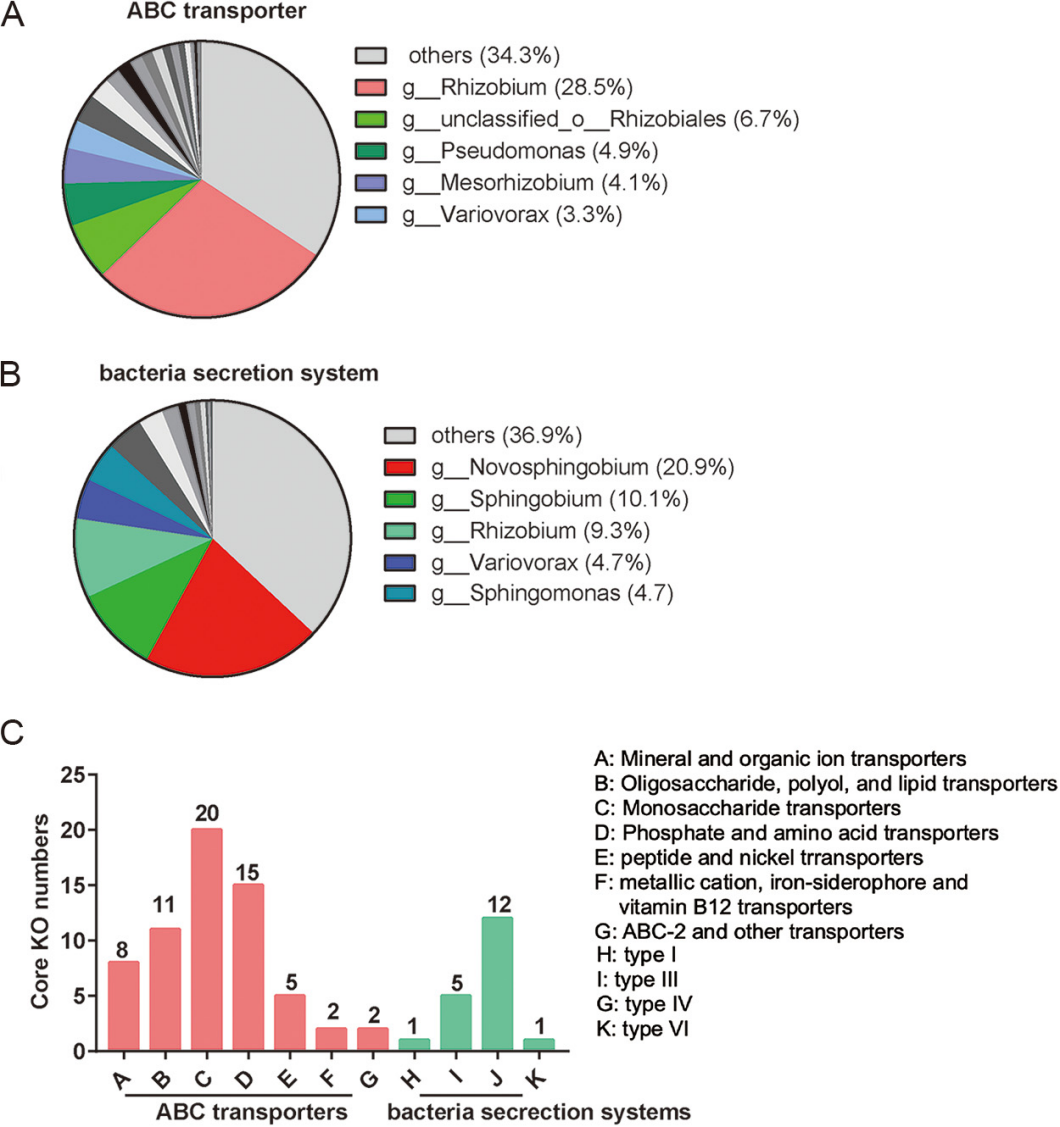
Linking the core functional traits and the species. (A and B) Species and functional contribution analysis. The relative abundance of a taxon participating in a function, calculated by summing the abundance of a taxon participating in the pathway assigned to that total abundance of all taxa involved in the function (the sum of the abundances of all taxa/functions was 1). The function contributed by a genus was determined as follows: (reads of the specific genus involved in the function)/(total reads of all taxa in the function). The relative contribution of different taxa (genus levels) is shown to identify the core functional attributes in pecan rhizosphere samples. (C) The distributions of the pecan rhizosphere core KOs in the ABC transporter and bacterial secretion system pathways.

The results indicate that *Rhizobium* participating in the ABC transporter function constituted up to 28.5% of the total abundance of all taxa involved in this function (Fig. 5A), while *Novosphingobium* participating in the bacterial secretion system constituted up to 20.9% (Fig. 5B), indicating that *Rhizobium* and *Novosphingobium* are the main contributors of the ABC transporter and the bacterial secretion system, respectively. Most of the core KOs in the ABC transporter were annotated as a monosaccharide transporter, followed by phosphate and amino acid transporters and oligosaccharide, polyol, and lipid transporters (Fig. 5C). These results suggest that the seedling pecan rhizosphere niche may contain a high abundance of monosaccharides. In addition, most of the core KOs in the bacterial secretion systems were annotated as the type IV secretion system (Fig. 5C), suggesting that this system plays an important role in shaping the composition of the pecan rhizosphere community.

## DISCUSSION

Plants consistently recruit certain taxa from the bulk soil, which play critical roles in plant nutrition and health (32). In this work, we performed a comparative study of the taxonomic and functional traits of microbiomes in the bulk soil and rhizosphere soil of the two nut trees at the seedling stage. Our findings reveal a significant difference in the community composition between the rhizosphere and the bulk soil, with a higher number of enrichment and depletion bacteria or KOs observed in the pecan rhizosphere than that in the hickory rhizosphere (Fig. 2C and D). These results indicate that the seedling pecan has a strong capacity to shape its rhizosphere microbiome, which may play a critical role in pecan health. Interestingly, it was reported that phylogenetically closely related plants might show similar selectivity for rhizosphere bacteria (33). However, we found that pecan and hickory showed different rhizosphere microbiomes (Fig. 2A). We therefore reasoned that significant enrichment bacteria in the seedling pecan rhizosphere probably assist the plant in overcoming the environmental challenges, as pecan exhibits higher resistance to multiple abiotic stresses than hickory (21).

*Rhizobium*, a plant-growth-promoting rhizobacterium, is capable of producing indole acetic acid and gibberellic acid, which are growth hormones for germination and plant growth (34). Previous research has demonstrated that inoculating maize with Rhizobium tropici can mitigate salinity stress (35). Interestingly, we found that *Rhizobium* is the most abundant genus in the rhizosphere (Fig. 3C) of the pecan, which is a known saline-alkali-tolerant plant (24). This finding suggests that recruiting *Rhizobium* might be a useful strategy for the pecan to use to resist saline stress. *Sphingomonas*, *Sphingobium*, and *Novosphingobium*, the rhizoremediation strain (36), hold great potential for the remediation of contaminated soil (37). In this study, interestingly, these strains were not only found to be the core genera of pecan but also constituted the most abundant genera in its rhizosphere (Fig. 3C). Our results suggest that these bacteria were probably recruited by the pecan to help remediate contaminated soil. *Rhizobium* and *Sphingomonas* were the core bacteria that were identified in other plant species, such as those of citrus (18) and grapevine (17). Moreover, we found that 76.22% of the core genera of pecan belong to *Proteobacteria* (Fig. 3B), which is a copiotroph phylum adapted to carbon-rich conditions (common in the rhizosphere) for high metabolic activity, fast growth, and propagation (38, 39). This phylum is commonly found in diverse plant species (40).These findings suggest that the presence of certain core microbial taxa may be common across plant species (3) and that the factors recruiting *Rhizobium* and *Sphingomonas* in pecan might be similar to those in other plant species. Most fruit tree crops are composed of the following two parts: the aboveground fruit-bearing part (the scion) and the below ground part (the rootstock), which provides anchorage and is responsible for water and nutrient uptake. The rootstocks could maximize benefits from rhizobiomes (41), which help plants to resist disease and tolerate the abiotic stresses. Thus, the core of rhizosphere microbes on pecan may represent candidate taxa for designing microbial consortia with a potential to serve as biofertilizers (42) and the identification of the core microbes provides a useful starting point for future studies that could exploit synthetic communities to determine the interaction between microbes in their interactions with pecan itself.

Deciphering how plants recruit the core rhizosphere bacteria is of great importance to understand plant-microbiome interactions. Plants shape the rhizosphere microbiome to skillfully utilize the microbial functional repertoire (43), which is closely associated with the life activities of the microorganism’s host-microbe and microbe-microbe interactions. For example, the ABC transporters play an important role in transporting ions, amino acids, monosaccharides, and oligosaccharides into microbial cells. Bacteria use a secretion system to communicate with their host and other organisms through secreting macromolecules into the external environment (44). Here, we observed that the ABC transporters and bacterial secretion system were the core functional traits of the pecan rhizosphere (Fig. 4B and C). The results suggest that the coevolution of host-microbe and microbe-microbe interactions is logically linked to the conditions present in the rhizosphere, thus accounting for their positive selection (45).

Plant-microbe and microbe-microbe interactions are important factors that influence the assembly of rhizosphere microbiomes. Through our analysis of the core functional traits and the species, we found that *Rhizobium* and *Novosphingobium* were the main contributors to the ABC transporter and the bacterial secretion system, respectively (Fig. 5A and B). Furthermore, we observed that the KOs that encoded the myo-inositol/inositol 1-phosphate transport were greatly enriched in the pecan rhizosphere bacteria (see Fig. S3 in the supplemental material). Interestingly, almost all KOs encoding type IV secretion system genes were specifically enriched in the pecan rhizosphere (see Fig. S4 in the supplemental material). Recent research has shown that myo-inositol serves as an alternative carbon source for various soil bacteria and plays an important role in mediating rhizobial symbiosis with legume plants (46, 47). Some bacteria deploy a type IV secretion system to deliver DNA, protein, or other macromolecules to bacterial or eukaryotic cell targets (48, 49). Our results, along with previous findings, suggest that myo-inositol may contribute to *Rhizobium* efficient colonization of the pecan rhizosphere. At the same time, the type IV secretion system might be used by *Novosphingobium* to communicate with the pecan and to interact with other bacteria in the pecan rhizosphere. These interactions probably trigger the physical response of the plant to resistant stresses and help to shape the composition of the rhizosphere community. These data provide insights into the mechanisms underlying the plant-mediated shaping of the rhizosphere microbiome.

Plants commonly release aromatic compounds, often as defenses to plant pathogens. Our study found that the functions involved in benzoate degradation, porphyrin and chlorophyll metabolism, and the xylene degradation pathway were enriched only in the pecan rhizosphere but not the hickory rhizosphere (Fig. 4C). Thus, this finding suggests that releasing aromatic compounds may be a strategy for the disease resistance of the pecan. Moreover, certain plant exudates and stable pollutants, such as polycyclic aromatic hydrocarbon, benzoate, and chlorophenols, can be degraded by some rhizosphere microbes, resulting in a rhizoremediation (37). Thus, the enrichment of the degradation pathways in the pecan rhizosphere suggests that the rhizosphere bacteria, such as *Sphingomonas*, *Sphingobium*, and *Novosphingobium*, likely degrade potential exogenous pollutants to reduce the autotoxicity of seedling pecan.

In conclusion, our data indicate that (i) the seedling pecan has a strong selective power in shaping the rhizosphere bacterial community. Some plant-beneficial bacteria are efficiently recruited by pecan, which may be an important strategy for pecan resistance to multiple abiotic stresses. This information relates to isolating and culturing the functional microbes, potentially harnessing the power of the microbiome to improve hickory health and production. (ii) The functional traits of ABC transporters (e.g., monosaccharide) and bacterial secretion systems (e.g., the type IV secretion system) are significantly abundant in the seedling pecan rhizosphere bacteria. These traits were mainly contributed by *Rhizobium* and *Novosphingobium*. This result suggests that some root exudates, such as monosaccharide, may help *Rhizobium* efficiently colonize the rhizosphere and the type IV secretion system might be used by *Novosphingobium* to shape the composition of the pecan rhizosphere community.

## MATERIALS AND METHODS

### Sample collection.

Pecan (*Carya illinoinensis*) and Chinese hickory (*Carya cathayensis* Sarg) were grown in a closed-environment greenhouse to eliminate the effect of the environment. After the plants grew for 6 months, the seedlings were excavated from the soil with a shovel and the roots were gently shaken to remove the soil that was not tightly attached to the roots. The roots were pooled and washed three times with phosphate-buffered saline (PBS). The washed-off soil was poured into a 50-mL centrifuge tube, centrifuged, and stored at −80°C until DNA extraction. This soil was termed the rhizosphere compartment. Soil from the same 5- to 15-cm deep area in the greenhouse without any roots was also collected, stored at −80°C until DNA extraction, and was termed bulk soil. In total, 10 rhizosphere soil samples and 6 bulk soil samples were obtained, with *n* = 5 for the rhizosphere group and *n* = 3 for the bulk soil group.

### DNA extraction and sequencing.

DNA was extracted from each sample using a Powersoil DNA extraction kit (Qiagen, USA) following the manufacturer’s instructions. The DNA quality and quantity were determined using a NanoDrop spectrophotometer (Thermo Scientific, Wilmington, DE). The DNA samples were stored at −80°C. For metagenomic library preparation, extracted DNA was fragmented to an average size of about 400 bp using a Covaris M220 instrument (Gene Company, China) for paired-end library construction. The paired-end library was constructed using a Nextflex Rapid DNA-seq kit (Bio Scientific, USA). Adapters containing the full complement of sequencing primer hybridization sites were ligated to the blunt end of fragments. Paired-end sequencing was performed using a HiSeq instrument (Illumina Inc., USA) at Majorbio in Shanghai, China.

### Metagenomic data analysis.

The data were analyzed on the online Majorbio cloud platform (http://www.majorbio.com). Briefly, the paired-end Illumina reads were trimmed of adaptors, and low-quality reads (length of <50 bp, with a quality value of <20 or having N bases) were removed by Fastp (50) (https://github.com/OpenGene/fastp; version 0.20.0). Metagenomics data were assembled using MEGAHIT (51) (https://github.com/voutcn/megahit; version 1.1.2), which makes use of succinct de Bruijn graphs. Contigs with a length of ≥300 bp were selected as the final assembling result, and then the contigs were used for gene prediction and annotation. Open reading frames (ORFs) from each assembled contig were predicted using Prodigal (52) (https://github.com/voutcn/megahit; v1.1.2). The predicted ORFs with a length of ≥100 bp were retrieved and translated into amino acid sequences using the NCBI translation table (http://www.ncbi.nlm.nih.gov/Taxonomy/taxonomyhome.html/index.cgi?chapter=tgencodes#SG1). Nonredundant gene catalogs were constructed using CD-HIT (53) (http://www.bioinformatics.org/cd-hit/; version 4.6.1) with 90% sequence identity and 90% coverage. High-quality reads were aligned to the nonredundant gene catalogs to calculate gene abundance with 95% identity using SOAPaligner (54) (https://github.com/ShujiaHuang/SOAPaligner; version soap 2.21). The taxonomic annotation was performed based on the nonredundant gene set. Representative sequences of the nonredundant gene catalog were aligned to the nonredundant (NR) database with an E value cutoff of 1e-5 using Diamond (55) (https://github.com/bbuchfink/diamond) for taxonomic annotations. To obtain functional information for the unigene, the protein sequences were homology searched against the KO database using Diamond software with an E value cutoff of 1e-5.

### Data analysis.

The beta-diversity analysis included a principal coordinate analysis (PCoA) and nonmetric multidimensional scaling (NMDS) analysis on Bray-Curtis distance through the online tool at the Majorbio cloud platform (https://cloud.majorbio.com/page/tools/). Differences in community composition among *C. cathayensis*-rhizo versus *C. cathayensis*-bulk, *C. illinoinensis*-rhizo versus *C. illinoinensis*-bulk, and *C. cathayensis*-bulk versus *C. illinoinensis*-bulk groups were tested by permutational analysis of variance (PERMANOVA). The Venn diagram plots were drawn using the Venn Diagram package; enrichment analysis of the Kyoto Encyclopedia of Genes and Genomes (KEGG) was carried out based on the cluster Profiler R package (56). KEGG terms with a *P*_adjust_ value of <0.05 were considered and plotted.

### Comparative analysis.

Based on the abundance profiles, the features (genera, phyla, and KOs) with significantly different abundances across compartments were determined using DESeq2 through the online tool at the Majorbio cloud platform (https://cloud.majorbio.com/page/tools/). For the detection of rhizosphere-enriched genera (abundance significantly higher than that in the bulk soil), rhizosphere-depleted genera (abundance significantly lower than that in the bulk soil), and the enriched or depleted KO of the rhizosphere groups in the metagenomic data, a paired DESeq2 comparison analysis was performed based on the read count matrix of the genera and KOs across the bulk soil and rhizosphere samples (*n* = 5 for the rhizosphere group, *n* = 3 for the bulk soil group). *P* values for multiple testing were corrected using the Benjamini and Hochberg (BH) method in DESeq2. All items with adjusted *P* values of <0.05 were considered significant. Furthermore, core pecan rhizosphere microbial genera and core pecan rhizosphere KOs were defined based on the genera or KOs that were both enriched in the pecan rhizosphere compared with those in the corresponding bulk soil and in the hickory rhizosphere. The relative abundances of rhizosphere-enriched or rhizosphere-depleted taxa and functional traits were visualized using the volcano plot package in R software.

### Species and functional contribution analysis.

The analysis of linking the core functional traits and the species was conducted as described previously (57) with the following modifications. The relative contribution of taxa to the core function, that is, the taxonomic information for each selected gene (i.e., the genes associated with the ABC transporter and the bacteria secretion system), in the pecan rhizosphere samples was extracted. Then, the functional contributions of observed microbial taxa (genus level) to the functional pathways were explored using customized R scripts. The relative contribution of a taxon was represented by the relative abundance of a taxon participating in a function, which was calculated by summing the abundance of a taxon participating in the function assigned to that total abundance of all taxa involved in that function. Therefore, the sum of the abundances of all taxa/function detected in each subject was 1.

### Data availability.

The data sets generated for this study can be found in the raw sequences of the metagenome that are available in the NCBI Sequence Read Archive under (BioProject PRJNA917788) with the accession number SRP416937.
