# Supplementary material for: Exploring the Core Bacteria and Functional Traits in Pecan (Carya illinoinensis) Rhizosphere
Source: Microbiol Spectr. 2023 Jun 13;11(4):e00110-23. doi: 10.1128/spectrum.00110-23 (PMC10433825; doi:10.1128/spectrum.00110-23)
Supplement: Supplemental file 10 — Supplemental material. Download spectrum.00110-23-s0010.pdf, PDF file, 0.3 MB [file spectrum.00110-23-s0010.pdf]

Table s1

Permanova test of NR

Characteristics	SumsOfSqs	MeanSqs	F.Model	R2	P-value	P.adjust
C.ca -rhizo VS bulk soil-C.ca-bulk	0.19515	0.19515	12.50905	0.67583	0.018	0.018
C.il-rhizo VS C.cil-bulk	0.08278	0.08278	6.96526	0.53722	0.013	0.013
C.ca-rhizo VS C.il-rhizo	0.1166	0.1166	6.39736	0.44434	0.009	0.009
C.ca-bulk VS C.cil-bulk	0.007	0.007	1.46483	0.26805	0.3	0.3

Table s2

Permanova test of KO

Characteristics	SumsOfSqs	MeanSqs	F.Model	R2	P-value	P.adjust
C.ca -rhizo VS C.ca-bulk	0.00822	0.00822	6.20677	0.55384	0.056	0.056
C.il-rhizo VS C.il-bulk	0.0223	0.0223	10.95731	0.64617	0.015	0.015
C.ca-rhizo VS C.il-rhizo	0.0089	0.0089	3.96197	0.33121	0.014	0.014
C.ca-bulk VS C.cil-bulk	0.0017	0.0017	1.67505	0.29516	0.2	0.2

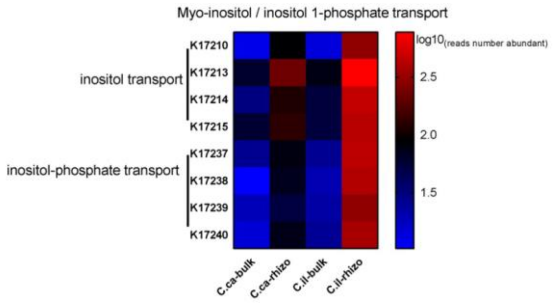

Figure S3 Myo-inositol/inositol 1-phosphate transport enriched in the pecan rhizosphere bacteria A heat map of abundances of the KOs involved in Myo-inositol/inositol 1-phosphate transport. The color from blue to red represents a relative abundance of each KO from low to high. C.il$_{\text{-Bulk}}$: *Carya.illinoinensis* (pecan)-bulk soil, C.il$_{\text{-rhizo}}$: *Carya.illinoinensis* (pecan)-rhizosphere soil, C.ca$_{\text{-Bulk}}$: *Carya. cathayensis Sarg* (hickory)-bulk soil and C.ca$_{\text{-rhizo}}$: *Carya cathayensis Sarg* (hickory)-rhizosphere soil.

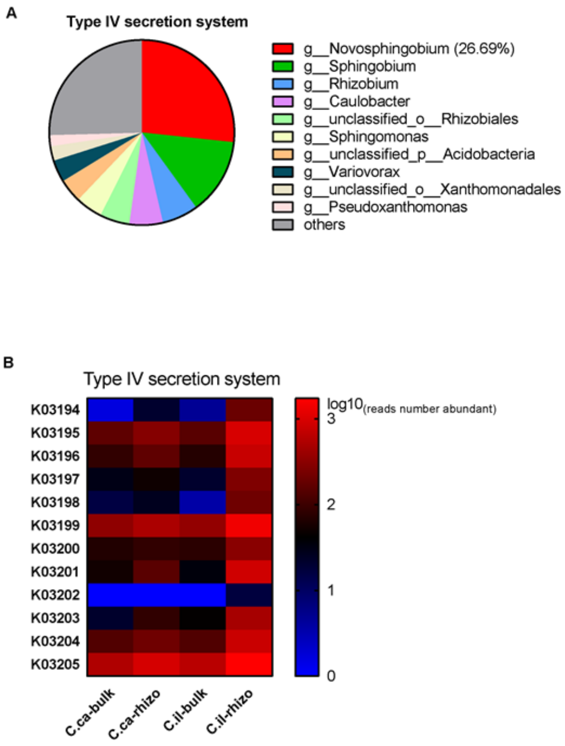

Figure S4 (A) Type IV secretion system was enriched in the pecan rhizosphere bacteria A heat map of abundances of the KOs involved in Type IV secretion system. The color from blue to red represents a relative abundance of each KO from low to high. **(B) Linking the type IV secretion system and the species** Species and functional contribution analysis. Relative contribution of different taxa (genera level) to identify the type IV secretion system attributes in pecan rhizosphere samples. The top ten genus was present. C.il$_{\text{-bulk}}$: *Carya.illinoinensis* (pecan)-bulk soil, C.il$_{\text{-rhizo}}$: *Carya.illinoinensis* (pecan)-rhizosphere soil, C.ca$_{\text{-bulk}}$: *Carya. cathayensis Sarg* (hickory)-bulk soil and C.ca$_{\text{-rhizo}}$: *Carya cathayensis Sarg* (hickory)-rhizosphere soil.
